# Bowman Layer Transplantation for Treating Keratoconus—Preliminary Findings

**DOI:** 10.3390/jcm12062402

**Published:** 2023-03-20

**Authors:** Eline Elodie Barbara De Clerck, Giorgio Enrico Bravetti, Martina Kropp, Horace Massa, Bojan Pajic, Gabriele Thumann, Ivo Guber

**Affiliations:** 1 Division of Ophthalmology, Department of Clinical Neurosciences, Geneva University Hospitals, 1205 Geneva, Switzerland; 2 Experimental Ophthalmology, University of Geneva, 1205 Geneva, Switzerland; 3 Eye Clinic ORASIS, Swiss Eye Research Foundation, 5734 Reinach, Switzerland; 4 Faculty of Sciences, Department of Physics, University of Novi Sad, Trg Dositeja Obradovica 4, 21000 Novi Sad, Serbia; 5 Faculty of Medicine of the Military Medical Academy, University of Defense, 11000 Belgrade, Serbia

**Keywords:** keratoconus, Bowman layer, transplantation

## Abstract

(1) Background: Mid-stromal isolated Bowman layer transplantation aims to reduce and stabilize corneal ectasia in patients with advanced, progressive keratoconus. The purpose of this review is to evaluate the effectiveness and safety of this new surgical technique. (2) Methods: Following the PRISMA statement and checklist, we searched Medline, the Cochrane Controlled Trials Register, and Embase and used a broad systematic search strategy according to the Cochrane Collaboration. (3) Results: Eight studies with a total number of 120 eyes of 106 patients met our inclusion criteria. One month after Bowman layer transplantation, patients with keratoconus showed a significant decrease in the measured simulated keratometry (−4.74 D [95% CI −6.79 to −2.69]) and the maximum keratometry (−7.41 D [95% CI −9.64 to −5.19]), which remained significant one year postoperatively (−2.91 D [95% CI −5.29 to −0.53] and −5.80 D [−8.49 to −3.12]). Intra- and postoperative complications were observed in 3% and 9% of the patients, respectively. An estimated success rate of 75% to 85% was achieved by experienced surgeons at 5 to 8 years postoperatively. (4) Conclusions: Bowman layer transplantation may be an effective and safe treatment option in patients with advanced, progressive keratoconus. Additional multicenter prospective interventional studies are needed to confirm these preliminary findings.

## 1. Introduction

Keratoconus is a bilateral, asymmetric, and often progressive protrusion and thinning of the cornea that results in high and irregular astigmatism, compromising visual function [1,2]. The overall prevalence rate is approximately 55 per 100,000 people [3]. The exact contribution of genetic, environmental, mechanical, and inflammatory factors remains unclear [4,5,6]. Different grading systems for classifying keratoconus have been proposed, e.g., the Amsler–Krumeich classification system, which is based on refraction, central keratometry, pachymetry, and the presence of corneal scarring, or the ABCD grading system, which also includes visual acuity [7,8,9].

The clinical presentation of keratoconus depends on disease severity (Table 1). According to the keratoconus severity and visual demands of the patient, several treatments are available. Early treatment options include the use of spectacles or soft contact lenses. In moderate cases, specially designed soft contact lenses, hybrid, rigid gas-permeable contact lenses, or scleral lenses are indicated [10].

**Figure 1:**
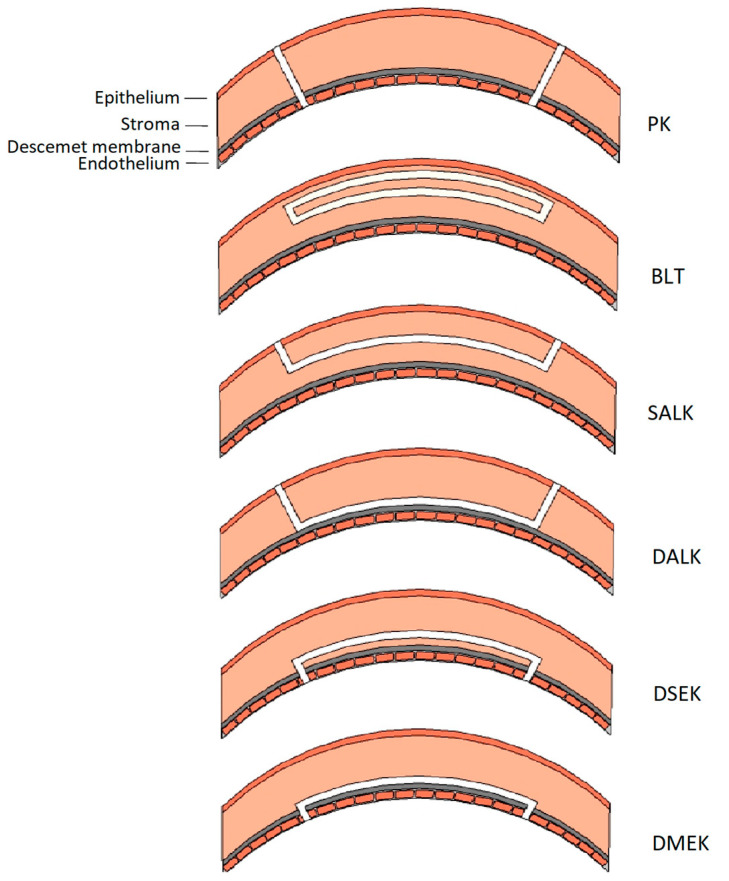
Schematic representation of the different types of keratoplasty. The white section represents the transplanted tissue. PK = penetrating keratoplasty. BLT = Bowman layer transplantation. SALK = superficial anterior lamellar keratoplasty. DALK = deep anterior lamellar keratoplasty. DSEK = Descemet stripping endothelial keratoplasty. DMEK = Descemet membrane endothelial keratoplasty.

In advanced stages with low visual acuity or contact lens intolerance, penetrating keratoplasty (PK), or deep anterior lamellar keratoplasty (DALK), remains the gold standard (Figure 1) [11,12]. The outcomes of these corneal graft surgeries are good, but postoperative complications related to sutures, epithelial wound healing, intraocular infections, graft rejection, peripheral keratoconus progression, or recurrent disease in the donor button are reported [13,14]. These complications can be particularly challenging in patients with coexistent atopic disease or ocular surface disorders [15,16]. Furthermore, young patients may need re-grafting at a later age, which is known to have less-favorable clinical outcomes [17].

To postpone the need for corneal transplantation in patients with progressive keratoconus with rather good vision, UV-induced collagen cross-linking may be indicated [18]. Moreover, intracorneal ring-segment implantation can be considered to improve vision and/or contact lens tolerance [19,20,21]. However, both treatment options are not advised in eyes with severe corneal thinning (<350 μm) and steepening (>58 D), as in advanced keratoconus [22,23,24,25,26]. Since each treatment option has side effects and limitations, the objective evaluation of the effectiveness and safety of new minimally invasive surgical techniques is paramount.

Bowman layer transplantation (BLT) is considered a promising alternative treatment in advanced, progressive keratoconus and can prevent most of the clinical challenges related to PK or DALK [27,28]. Since fragmentation and thinning of the Bowman layer are characteristics of advanced keratoconus [29], mid-stromal implantation of a donor Bowman layer could partially restore corneal anatomy and slow down or arrest the progression of the disease [27].

Donor tissue preparation for BLT consists of manually peeling the Bowman layer from the anterior stroma of a whole donor globe or a donor corneoscleral rim, which is mounted on a globe holder or an artificial anterior chamber [30]. Next, the epithelium is carefully debrided using surgical spears, and a superficial circular incision with a diameter of 9 to 11 mm is made within the limbal corneal periphery using a 30-gauge needle. Then, a McPherson forceps or custom-made tying forceps with round edges is used to lift and grasp the peripheral Bowman layer edge and peel the Bowman layer away from the underlying anterior stroma. Finally, the graft is submerged in 70% ethanol to remove any remaining epithelial cells, rinsed with BSS, and stored in organ culture medium before transplantation [27].

The first steps of the surgical technique resemble manual DALK surgery during which a stromal pocket is dissected over 360 degrees up to the limbus within the recipient cornea using an air bubble in the anterior chamber as a reference plane to judge the depth of dissection [27,31,32]. This dissection can be performed manually or with the assistance of a femtosecond laser [28,33,34]. In contrast with DALK surgery, the intended depth is 50% instead of 99%, allowing transplantation in very thin corneas and reducing the risk of intra-operative corneal perforation [11]. Air is then removed from the anterior chamber. Next, the Bowman layer graft is rinsed with BSS, stained with trypan blue, inserted through the scleral tunnel into the stromal pocket with or without the help of a glide, and stretched out to the corneal periphery and centered. Finally, the anterior chamber is re-pressurized with BSS. Postoperative medication includes topical Chloramphenicol 0.5% and dexamethasone 0.1%, followed by fluorometholone 0.1% tapering [27].

BLT aims to maintain functional visual acuity, preserve a patient’s corneal tissue, and delay or avoid more invasive surgeries such as PK or DALK while reducing the risk of postoperative complications [27]. These effects are reported as being stable with no significant differences after 6 to 18 months’ follow-up [28,32,35]. The Bowman layer graft induces a flattening of the cornea by pulling its anterior surface, which directly reduces the spherical aberration [27,35]. Intracorneal ring-segment implantation (polymethylmethacrylate) also induces this effect but the risk of migration and interface reaction is much lower for BLT. This is due to the similar mechanical characteristics of this tissue with the surrounding corneal stroma [27]. Furthermore, the risk of allograft rejection is considered negligible since the Bowman layer consists of collagen fibers with no cellular material. Flattening of the cornea in advanced keratoconus (approximately 8 D) improves contact lens tolerance [28,31].

However, introducing irregular interfaces or a layer with a different refraction index can lead to backscattering-inducing glare and lower contrast sensitivity [36,37]. Furthermore, perforation of the host Descemet membrane can occur intra-operatively [31,32]. Additionally, the weakening of the stroma due to the Bowman layer insertion can result in the accumulation of fluid in the form of “fluid lake-like hypodense areas” or hydrops. However, spontaneous resorption of hydrops is reported. Eye rubbing is identified as a risk behavior [37,38].

The aim of this review is to systematically investigate the effectiveness and safety of BLT as a selective, minimally invasive treatment for patients with advanced keratoconus. This is important given the novel characteristics of this treatment and the lack of large multicenter studies.

## 2. Materials and Methods

### 2.1. Search Strategy and Selection Criteria

Following the PRISMA statement and checklist (Appendix D) [39,40], we searched Medline, the Cochrane Controlled Trials Register, and Embase and used a broad systematic search strategy according to the Cochrane Collaboration (Appendix C). We searched for articles published up to 29 November 2022 using the terms “keratoconus”, “Bowman membrane”, and “corneal transplantation” without any language restrictions or limitations. The bibliographies of the included articles were screened until no new articles were found.

We included randomized controlled trials, cohort studies, prospective and retrospective case series, and case-control studies of adults (≥18 years) with keratoconus. Included studies had to present one or more outcome measures of mid-stromal isolated BLT. The assessed outcome variables included the (1) best spectacle-corrected visual acuity (logMAR), (2) best contact lens-corrected visual acuity (logMAR), (3) pachymetry thinnest point (µm), (4) pachymetry central point (µm), (5) maximum keratometry (D), and (6) mean simulated keratometry (D).

Author Eline De Clerck (EDC) selected the eligible studies and author Ivo Guber (IG) checked the selection. Study selection was carried out in two stages. First, we screened papers by reading the title, abstract, and keywords. We excluded reviews, letters, and comments. Second, we screened the full text of eligible papers and included them if they assessed one or more of the preselected postoperative outcome measures. Studies were excluded if they included patients with non-keratoconus ectasia, e.g., post-laser-assisted in situ keratomileusis (LASIK), if they used Bowman layer-only grafting, or if they used a Bowman-stromal inlay.

### 2.2. Data Extraction and Analysis

EDC reviewed the studies for inclusion and quality and extracted the pertinent clinical data. The data extraction sheet was based on the Cochrane Costumers and Communication Review Group’s data extraction template [41]. I.G. checked the data. Disagreements were resolved through discussion between the two review authors. Studies were not blinded with regard to the journal or any other aspect of the journal. The data extracted were the authors and year of publication, type of study, study design, country, number of patients with keratoconus, subgroups, inclusion and exclusion criteria, intervention (i.e., donor tissue, graft size, surgical technique, and postoperative medication), and the outcome variables studied, along with their mean values and standard deviations (SD). In addition, the age, sex, keratoconus stage, and the presence of corneal scarring were extracted.

Methodological quality was assessed according to the Delphi list [42] with one additional item. Table 2 describes the five quality items that were assessed. These domains were assessed by a score of “Yes” (high quality), “No” (low quality), or “Unclear” (uncertain quality).

The risk of bias was assessed according to the Cochrane guidelines [44]. Four domains were assessed: (1) Were the data collectors masked with respect to the identity of and medical results of the patients (performance bias)? (2) Were the outcome assessors masked with respect to the identity and medical results of the patients (detection bias)? (3) Were the reports of the study free of selective outcome reporting (reporting bias)? (4) Was the study free of other factors that could put it at risk of bias (selection bias, attrition bias, or other bias)? These domains were assessed by a score of “Yes” (low risk of bias), “No” (high risk of bias), or “Unclear” (uncertain risk of bias).

All pooled analyses were based on random-effects models because of the differences between the included studies in terms of the study population, intervention, and outcomes [45]. Statistical analyses were performed with Microsoft^®^ Excel^®^ 2016 MSO (Version 2302 Build 16.0.16130.20186) and Review Manager version 5.4.1.

The mean preoperative outcome variables of patients with keratoconus were compared with the postoperative outcome variables assessed 1 month, 6 months, and 1 year postoperatively to assess the performance of BLT. Next, the complications of BLT were assessed. Finally, the success rate was evaluated.

The changes in the outcome variables were assessed with the summary point estimates from the random-effects meta-analyses and 95% CIs [46]. Negative values indicate that the outcome variable decreased in individuals after BLT surgery compared with the same outcome variable measured preoperatively. Heterogeneity between studies was addressed with a statistical Χ^2^ and I^2^ test (Χ^2^ test: *p* < 0.05; I^2^ test ≥30%) [47].

## 3. Results

### 3.1. Selected Studies

**Figure 2:**
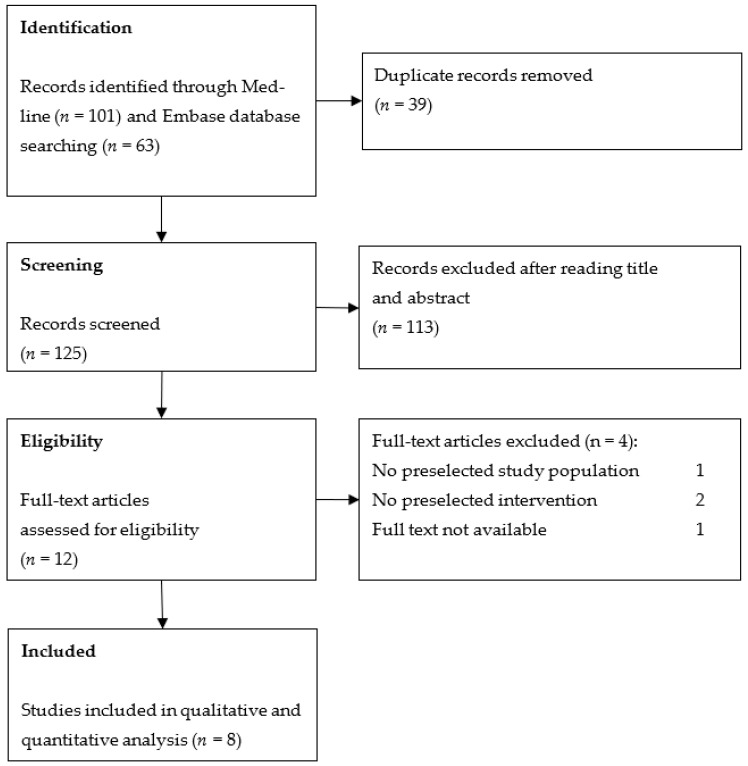
Flow diagram of study selection.

A total of 164 articles were identified through database searching. After removing duplicates, 125 articles were screened. Only 12 articles assessed BLT in patients with keratoconus. Finally, eight studies met our inclusion criteria (Figure 2).

The characteristics of the included studies are shown in Table 3 and Appendix B. Six studies were retrospective case series [27,28,32,35,36,43], one study was a prospective case series [37], and one study was a prospective cohort study [31].

### 3.2. Quality Assessment

Information about the consecutiveness of the sample was insufficient in six out of the eight studies [27,31,32,35,37,43]. The selection criteria were heterogeneous among the studies. The studies included keratoconus stages II to IV (Table 3). One study excluded individuals with corneal opacities or healed hydrops [35] and one study excluded unsuccessful, complicated surgery [32]. All included studies reported point estimates and SDs for the outcome measures [27,28,31,32,35,36,37,43]. In one study, some of the outcome variables were only shown in box plots [36]. One study also reported outcome variables in the contralateral untreated eye [37].

The quality assessment of the included studies is shown in Appendix B. Table 2 shows the quality assessment questions used across the studies.

### 3.3. Risk of Bias in Included Studies

The risk of bias among the included studies is presented in detail in Appendix B. In none of the studies were the data collectors or the outcome assessors masked. In one of the studies, the presence of selective reporting was unclear [28]. All studies seemed to be subject to other sources of bias due to the relatively small sample size for the published studies and the absence of a control group.

### 3.4. Outcome Analyses and Investigation of Heterogeneity

Table 3 shows the baseline characteristics of the study population of the included studies. Eight studies with a total of 120 eyes of 106 patients with a mean age of 30 years within an age range of 7–71 years, including at least 51 males, met our inclusion criteria. According to the Amsler–Krumeich classification, one study included keratoconus stages II-IV [43], five studies included keratoconus stages III-IV [31,32,35,36,37], and two studies only included end-stage keratoconus [27,28].

Table 4 shows the numerical data for the outcome variables. The graphical data and the Χ^2^ and I^2^ values for heterogeneity are shown in full in Appendix A.

One year postoperatively, patients who underwent BLT had a significantly higher best spectacle-corrected visual acuity [−0.37 logMAR, 95% CI (−0.62 to −0.12), *p* < 0.01] and a significantly higher central point pachymetry [+24.39 µm, 95% CI (+0.28 to +48.49), *p* = 0.05]. Maximum keratometry was significantly decreased in patients 1 month [−7.41 D, 95% CI (−9.64 to −5.19), *p* < 0.001], 6 months [−6.90 D, 95% CI (−9.27 to −4.52), *p* < 0.001], and 1 year postoperatively [−5.80 D, 95% CI (−8.49 to −3.12), *p* < 0.001] compared to preoperative values. The mean simulated keratometry values were also significantly decreased in patients 1 month [−4.74 D, 95% CI (−6.79 to −2.69), *p* < 0.001], 6 months [−4.79 D, 95% CI (−7.11 to −2.48), *p* = 0.01], and 1 year postoperatively [−2.91 D, 95% CI (−5.29 to −0.53), *p* = 0.02] compared to preoperative values.

We found no heterogeneity in the estimates reported, except for the difference in the best spectacle-corrected visual acuity (I^2^ test = 67%) and best contact lens-corrected visual acuity (I^2^ test = 30%) assessed one year postoperatively.

### 3.5. Complications and Success Rate

One hundred and three eyes were included in the assessment of the complications and success rate after the exclusion of duplicate data [31] and the inclusion of all complicated surgeries [35]. The mean follow-up time was 28 months (range of 3–60 months).

Intra- and postoperative complications were, respectively, observed in 3% and 9% of patients. Intraoperative perforation of the Descemet membrane occurred in 3% of eyes (N = 3), with a subsequent PK reported in 1% of eyes (N = 1) [32,37].

Six percent of eyes (N = 6) presented with acute hydrops at 43 months to 82 months postoperatively [37,43]. After topical treatment with dexamethasone eye drops and NaCl 5% ointment, corneal clearance with some residual scarring appeared in all eyes. Two percent of eyes (N = 2) presented with mild contact lens-related keratitis, one at 9 months and one at 75 months postoperatively [43]. One percent of eyes (N = 1) presented with a contact lens-related pseudomonas corneal ulcer at 54 months postoperatively (N = 1) [43].

Eight percent of eyes (N = 8) showed postoperative keratoconus progression [32,35,37,43]. One percent of eyes (N = 1) needed Bowman layer re-transplantation due to an unsatisfactory visual acuity result at 22 months postoperatively [43]. PK after intraoperative perforation of the Descemet membrane was needed in 1% of eyes (N = 1) [37]. If success for BLT is defined as the absence of postoperative keratoconus progression and the absence of re-transplantation, the estimated success rate varied between 75% and 85% at 5 to 8 years postoperatively (Kaplan–Meier analysis) [37,43].

## 4. Discussion

In this systematic review, we summarized the effectiveness and safety of mid-stromal isolated BLT in patients with keratoconus. To the best of our knowledge, this is the first systematic review to evaluate this new minimally invasive treatment option for patients with advanced keratoconus who are not eligible for UV-cross-linking or intracorneal ring-segment implantation, i.e., patients with eyes with severe corneal thinning and steepening [22,23,24,25,26]. This selective surgical technique aims to stabilize the corneal ectasia through the firmness of the graft and the wound-healing response [27,28]. Furthermore, the acellular nature of the Bowman layer graft typically eliminates the risk of allograft rejection.

The isolated Bowman layer graft can be prepared from a whole donor globe or a corneoscleral rim with equivalent success [30]. The latter preparation technique allows tissue economy since the remaining tissue can be re-used for endothelial grafts. The surgery itself consists of an extra-ocular technique, as the eye is never completely entered [11]. Thanks to the mid-stromal position of the graft, sutures can be avoided and the ocular surface remains intact. The use of the same surgical technique in all the included studies further strengthens the results of our analyses.

We noted that one month after BLT, patients with keratoconus showed a significant decrease in the measured simulated keratometry of approximately 4.5 D and a significant decrease in the maximum keratometry of approximately 7.5 D. Flattening was particularly pronounced in advanced keratoconus cases with central cones and remained significant one year postoperatively [31]. Two longitudinal studies showed that these topographic results also remained stable up to 8 years after surgery [37,43]. Therefore, we can conclude that BLT yields a long-lasting, optically improved anterior curvature.

The best spectacle-corrected visual acuity showed an initial increase one year postoperatively, which remained stable up to 5 years after surgery [37]. The best contact lens-corrected visual acuity remained stable after BLT. In addition, corneal higher-order aberrations, especially spherical aberrations, decreased up to one year after BLT [36].

Unfortunately, a postoperative increase in backscattering-inducing glare and lower contrast sensitivity has been described up to 5 years after BLT [36,37]. This could be explained by the mid-stromal position of the Bowman layer graft, which introduced interface irregularities or differences in refractive indices [36]. Additionally, the reliability of corneal densitometry and keratometry measurements has been questioned for patients with advanced keratoconus [48]. Therefore, further research on objective, repeatable, and reproducible measurements of vision quality is needed. Furthermore, a keratoconus classification system based on visual performance and corneal topometric and tomographic parameters would be useful [49].

According to our systematic review, intra- and postoperative complications are, respectively, reported in 3% and 9% of patients. Intraoperative perforation of the host Descemet membrane is a rare complication, which can either resolve spontaneously or require a re-transplantation. The postoperative risks of keratoconus progression and acute hydrops are notable, particularly in patients with a history of allergies or periocular atopy with postoperative eye rubbing.

Finally, an estimated success rate of between 75% and 85% at 5 to 8 years postoperatively was achieved by experienced surgeons [37,43]. This makes BLT a pertinent and safe minimally invasive treatment for patients with advanced, progressive keratoconus, and allows for the possibility of PK or DALK to be performed subsequently if needed.

Some methodological issues deserve discussion. Methods of the analysis and inclusion criteria were specified and documented in a protocol but this protocol was not prospectively registered. In addition, our results could be affected by publication bias, but this could not be assessed due to the small number of included studies.

Some issues at the study level also need to be addressed. Most of the studies included were retrospective case series. Most of them reported statistical power but the number of patients in several studies was small. However, the heterogeneity for the outcome variables was low.

The limited number of studies and study centers raises concerns about the performance of this treatment. These concerns could be dissipated by multicenter prospective studies.

Given the requirement for donor tissue and the relatively complicated surgical procedure, the cost effectiveness of BLT still needs to be evaluated [32,43].

## 5. Conclusions

BLT may be an effective and safe additional treatment option in patients with advanced, progressive keratoconus in order to postpone PK or DALK. However, large multicenter prospective interventional studies and longer follow-up data are needed to confirm these preliminary findings.

## Figures and Tables

**Table 1:** Treatment options.

Clinical Stage	Clinical Presentation	Treatment Options
Subclinical	First topographic changes visible	Spectacles, contact lenses, no eye rubbing
1	Simple evolutionary astigmatism	Spectacles, contact lenses, no eye rubbing, corneal cross-linking
2	Irregular astigmatism and myopia	Contact lenses, no eye rubbing, corneal cross-linking, intracorneal ring segment implantation, BLT
3	Deformation visible on slit lamp examination but cornea still clear	Contact lenses, no eye rubbing, corneal cross-linking (CCT > 400 μm), intracorneal ring-segment implantation, BLT
4	Important thinning with corneal scarring	DALK, PK

BLT = Bowman layer transplantation. CCT = central corneal thickness. DALK = deep anterior lamellar keratoplasty. PK = penetrating keratoplasty.

**Table 2:** Quality assessment.

Source [42]	Quality Item	No. of Publications Scored “Yes”
Added by authors	Consecutive patients?	2 [28,36]
Delphi list	Were inclusion criteria specified?	8 [27,28,31,32,35,36,37,43]
Delphi list	Were exclusion criteria specified?	2 [32,35]
Delphi list	Were point estimates and measures of variability presented for the primary outcome measures?	8 [27,28,31,32,35,36,37,43]
Considered for Delphi list	Was calculation of statistical power reported?	6 [27,31,35,36,37,43]

**Table 3:** Baseline characteristics of the study population.

Study	Number of Eyes/Patients	Age (Years)	Male Sex, *n* (%)	Keratoconus Stage	Eyes with Pre-Existing Corneal Scarring
Garcia de Oteyza 2019 [28]	2/2	-	-	IV	0
Luceri 2016 [36]	15/14	32 (17–71) *	6 (43)	III–IV	9
Shah 2022 [35]	11/11	18 (7)	8 (73)	III–IV	-
Tourkmani 2022 [32]	5/5	32 (21–40) *	-	III–IV	-
van der Star 2022 [43]	35/29	32 (13)	16 (55)	II–IV	19
van Dijk 2014 [27]	10/9	31 (16)	3 (33)	IV	-
van Dijk 2015 [31]	22/19	32 (13)	10 (53)	III–IV	12
van Dijk 2018 ** [37]	20/17	31 (12)	8 (47)	III–IV	12
Total	120/106	30 (7–71)	51	II–IV	

* Data are mean (SD), except for 2 studies giving the range; ** Previously reported data at follow-ups 1 month and 1 year postoperatively [31].

**Table 4:** Clinical outcome measures during follow-up visits compared with pre-operative values.

Clinical Outcome Measures	Postoperative Follow-Up		
	1 Month *	6 Months *	1 Year *
Best spectacle-corrected visual acuity (logMAR)	−0.08 [−0.23 to 0.06]	−0.21 [−0.46 to 0.05]	−0.37 [−0.62 to −0.12] **
Best contact lens-corrected visual acuity (logMAR)	0.16 [0.00 to 0.32] **	0.04 [−0.06 to 0.14]	0.07 [−0.01 to 0.15]
Pachymetry thinnest point (μm)	35.36 [8.40 to 62.32] **	17.00 [−13.51 to 47.51]	21.09 [−2.04 to 44.22]
Pachymetry central point (μm)	27.28 [−2.72 to 57.28]	15.45 [−19.23 to 50.13]	24.39 [0.28 to 48.49] **
Maximum keratometry (D)	−7.41 [−9.64 to −5.19] **	−6.90 [−9.27 to −4.52] **	−5.80 [−8.49 to −3.12] **
Mean simulated keratometry (D)	−4.74 [−6.79 to −2.69] **	−4.79 [−7.11 to −2.48] **	−2.91 [−5.29 to −0.53] **

* Data are mean effect size (95% CI). ** Significant decrease or increase in postoperative outcome measures.

**Figure A1:**
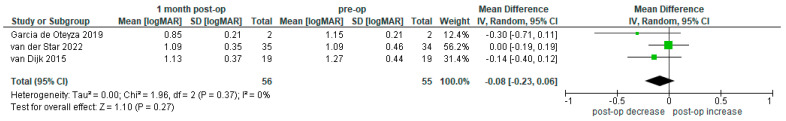
Difference in best spectacle-corrected visual acuity across 3 studies [28,37,43].

**Figure A2:**
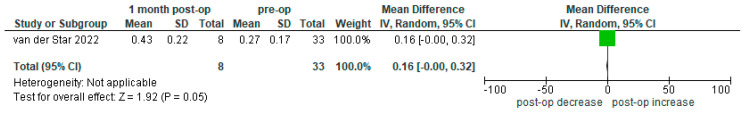
Difference in best contact lens-corrected visual acuity across 1 study [43].

**Figure A3:**
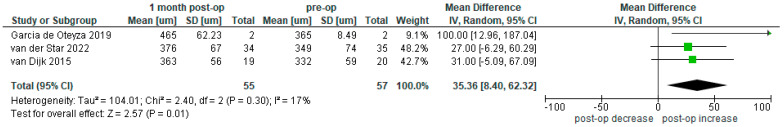
Difference in pachymetry thinnest point across 3 studies [28,31,43].

**Figure A4:**
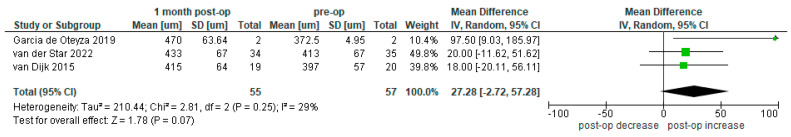
Difference in pachymetry central point across 3 studies [28,31,43].

**Figure A5:**
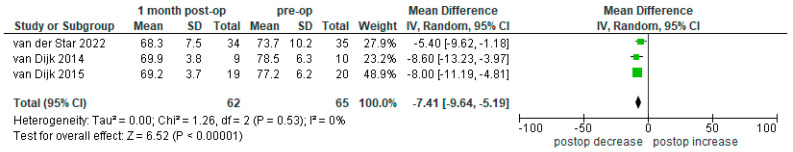
Difference in maximum keratometry across 3 studies [27,31,43].

**Figure A6:**
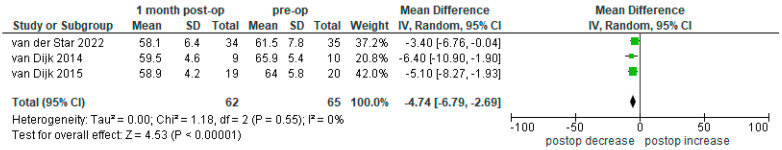
Difference in mean simulated keratometry across 3 studies [27,31,43].

**Figure A7:**
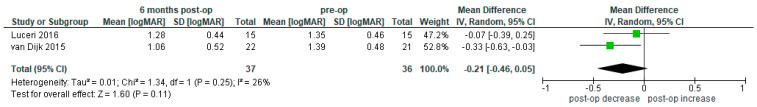
Difference in best spectacle-corrected visual acuity across 2 studies [31,36].

**Figure A8:**
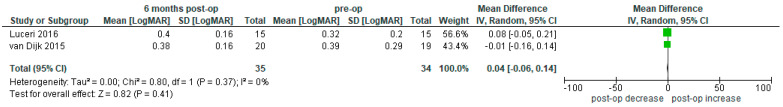
Difference in best contact lens-corrected visual acuity across 2 studies [31,36].

**Figure A9:**
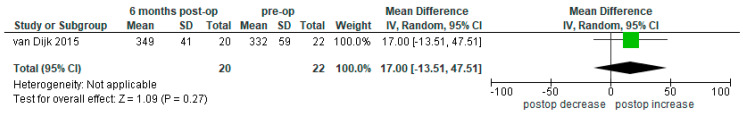
Difference in pachymetry thinnest point in 1 study [31].

**Figure A10:**
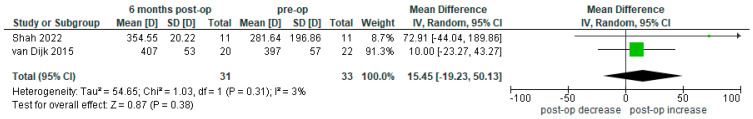
Difference in pachymetry central point across 2 studies [31,35].

**Figure A11:**
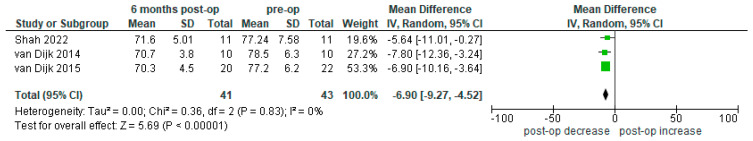
Difference in maximum keratometry across 3 studies [27,31,35].

**Figure A12:**
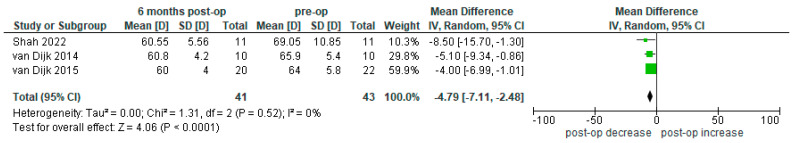
Difference in mean simulated keratometry in 3 studies [27,31,35].

**Figure A13:**
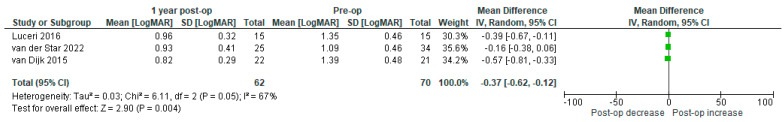
Difference in best spectacle-corrected visual acuity across 3 studies [31,36,43].

**Figure A14:**
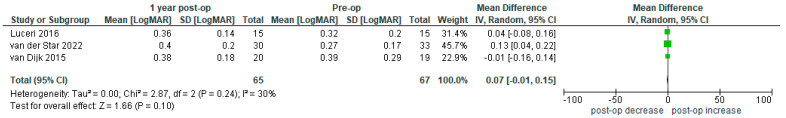
Difference in best contact lens-corrected visual acuity across 3 studies [31,36,43].

**Figure A15:**
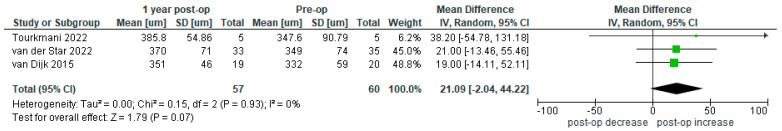
Difference in pachymetry thinnest point across 3 studies [32,37,43].

**Figure A16:**
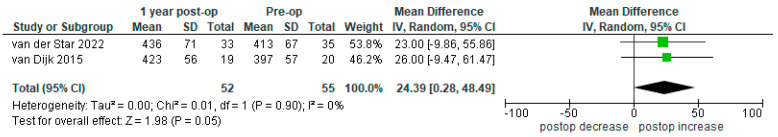
Difference in pachymetry central point across 2 studies [37,43].

**Figure A17:**
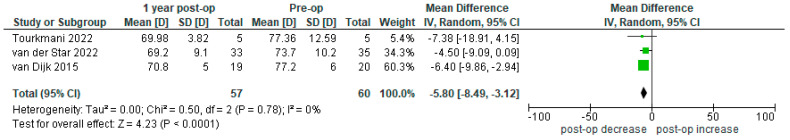
Difference in maximum keratometry across 3 studies [32,37,43].

**Figure A18:**
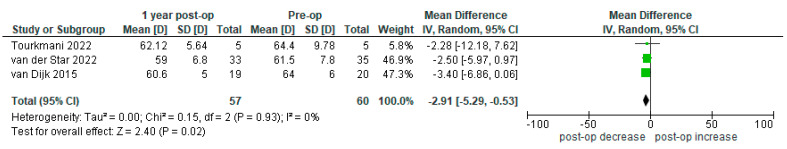
Difference in mean simulated keratometry across 3 studies [31,32,43].

## Data Availability

No new data were created.

## Appendix B. Characteristics of Included Studies

**Table A1:** Garía de Oteyza 2019 [28].

Methods	Type of study: Interventional study Study design: Retrospective case series
Participants	Country: Mexico Number of individuals with keratoconus: 2 Number of eyes: 2 Subgroups: preoperative, 1 month postoperative, 3 months postoperative Inclusion criteria: stage IV keratoconus (Amsler–Krumeich classification), contact lens/scleral lens intolerance, informed consent Exclusion criteria: -
Interventions	Donor tissue: donor cornea on artificial chamber (K20-215 Barron), epithelial removal, superficial incision, trypan bleu, Bowman layer peeling, saline solution Surgical technique: stromal pocket (femtosecond laser), dissection (SMILE spatula), Bowman layer over surgical glide, graft insertion into stromal pocket, centering Bowman layer (SMILE spatula)
Outcomes	UDVA (Snellen), CDVA (Snellen), CL tolerance, VA with CL (Snellen), K1 (D), K2 (D), CCT (μm), TCT (μm), complications, success rate, contact lens tolerance

**Table A2:** Quality assessment.

Item	Authors’ Judgement
Consecutive patients?	Yes
Reasons for inclusion reported?	Yes
Reasons for exclusion reported?	Unclear
Were point estimates and measures of variability presented for the outcome measures?	No: only 2 cases
Was calculation of statistical power reported?	No: only 2 cases

**Table A3:** Risk of bias.

Item	Authors’ Judgement
Data-collector blinded?	No
Outcome-assessor blinded?	No
Free of selective reporting?	Unclear
Free of other biases?	No: limited in number of patients

**Table A4:** Luceri 2016 [36].

Methods	Type of study: Interventional study Study design: Retrospective case series
Participants	Country: the Netherlands Number of individuals with keratoconus: 14 Number of eyes: 15 Subgroups: preoperative, 1 day postoperative, 1 week postoperative, 1 month postoperative, 3 months postoperative, 6 months postoperative, 12 months postoperative Inclusion criteria: advanced keratoconus with ectatic progression (simK 1D Δ ± Kmax 2D Δ), follow-up ≥ 1 year, aberrometric and densitometric data (software version 1.17 Pentacam) Exclusion criteria: -
Interventions	Donor tissue: artificial anterior chamber, epithelial removal, peeling Bowman layer (30G needle), ethanol 70% Surgical technique: scleral frown, mid-stromal pocket (Melles spatula set), glide (Visitec Surgical Glide), air removal, 70% ethanol, trypan blue, Bowman layer placement on the glide, graft insertion into stromal pocket, BSS in anterior chamber Postoperative medication: chloramphenicol eye drops 0.5% 6x/day (1 month), dexamethasone eye drops 0.1% 4x/day (1 month), followed by FML 4x/day, tapered to 1x/day over a period of 1 year
Outcomes	Anterior Km (D), anterior Kmax (D), Spectacle CDVA (LogMAR), CL CDVA (logMAR), pre-existing corneal scarring, depth of BL implantation (%), anterior and posterior higher-order aberrations, corneal densitometry zone 0–2 mm, zone 2–6 mm, zone 6–10 mm (anterior 120 μm, central layer, posterior 60 μm), subjective improvement

**Table A5:** Quality assessment.

Item	Authors’ Judgement
Consecutive patients?	Yes
Reasons for inclusion reported?	Yes
Reasons for exclusion reported?	Unclear
Were point estimates and measures of variability presented for the outcome measures?	Yes
Was calculation of statistical power reported?	Yes

**Table A6:** Risk of bias.

Item	Authors’ Judgement
Data-collector blinded?	No
Outcome-assessor blinded?	No
Free of selective reporting?	Yes
Free of other biases?	No

**Table A7:** Shah 2022 [35].

Methods	Type of study: Interventional study Study design: Retrospective case series
Participants	Country: PakistanNumber of individuals with keratoconus: 11 Number of treated eyes: 11 Number of untreated contralateral eyes: 11 Subgroups: preoperative, 6 months postoperative, 18 months postoperative Inclusion criteria: cornea thinnest ≥ 400 μm, Kmax > 58D, progressive keratoconus after UV cross-linking Exclusion criteria: corneal opacities, healed hydrops
Interventions	Donor tissue: artificial anterior chamber, epithelial removal (microsponge and spatula double-flap Buratto), vision blue, air injection in stroma, peeling Bowman layer (30G needle), ethanol 70%, BSS Surgical technique: scleral tunnel (6 mm length, 2 mm away from limbus), mid-stromal pocket (Melles spatula), glide (Visitec Surgical Glide), air removal, 70% ethanol, trypan blue, Bowman layer placement on the glide, graft insertion into stromal pocket, 30G canula and BSS in anterior chamber, cauterization conjunctival wound
Outcomes	Anterior K value (D), Kmax (D), posterior cornea back K value (D), corneal pachymetry (μm), progression of untreated contralateral eyes, contact lens tolerance

**Table A8:** Quality assessment.

Item	Authors’ Judgement
Consecutive patients?	Unclear
Reasons for inclusion reported?	Yes
Reasons for exclusion reported?	Yes
Were point estimates and measures of variability presented for the outcome measures?	Yes
Was calculation of statistical power reported?	Yes

**Table A9:** Risk of bias.

Item	Authors’ Judgement
Data-collector blinded?	No
Outcome-assessor blinded?	No
Free of selective reporting?	Yes
Free of other biases?	No

**Table A10:** Tourkmani 2022 [32].

Methods	Type of study: Interventional study Study design: Retrospective case series
Participants	Country: the United KingdomNumber of individuals with keratoconus: 5 Number of eyes: 5 Subgroups: preoperative, 1 year postoperative Inclusion criteria: keratoconus stage III-IV, contact lens intolerance Exclusion criteria: unsuccessful, complicated surgery
Interventions	Donor tissue: artificial anterior chamber, scoring mark (30G needle), scrape edge of scoring mark (McPherson forceps or Morlett spatula), peeling Bowman layer (Moorfield forceps) Surgical technique: direct introduction of the BL graft into the host corneal pocket (without glide) Graft size: 8 mm
Outcomes	Visual acuity, Kmax and Kmean (front cornea), keratometric values 4.5 mm and 6.0 mm (Holladay report), corneal cylinder (front cornea), corneal thickness at thinnest point, complications, contact lens tolerance

**Table A11:** Quality assessment.

Item	Authors’ Judgement
Consecutive patients?	Unclear
Reasons for inclusion reported?	Yes
Reasons for exclusion reported?	Yes
Were point estimates and measures of variability presented for the outcome measures?	Yes, except for corneal cylinder (SD missing)
Was calculation of statistical power reported?	No

**Table A12:** Risk of bias.

Item	Authors’ Judgement
Data-collector blinded?	No
Outcome-assessor blinded?	No
Free of selective reporting?	Yes
Free of other biases?	No

**Table A13:** Van Der Star 2022 [43].

Methods	Type of study: Interventional study Study design: Retrospective case series with prospectively collected data
Participants	Country: the Netherlands Number of individuals with keratoconus: 29 Number of eyes: 35 Subgroups: preoperative Kmax > 69D (group 1), preoperative Kmax < 69D (group 2), 1 month postoperative, 1 year postoperative, last available follow-up Inclusion criteria: keratoconus progression (Kmean ≥ 1D Δ ± Kmax ≥ 2D Δ), keratoconus stage II-IV Exclusion criteria: -
Interventions	Donor tissue: epithelial removal, superficial incision, lifting and grasping BL edge (McPherson forceps/custom-made tying forceps), peeling Bowman layer (30G needle), ethanol 70%, organ culture medium (CorneaMax) Surgical technique: air in anterior chamber, mid-stromal pocket, ethanol 70%, BSS, trypan blue 0.06%, Bowman layer placement on the glide (BD Visitec Surgical Glide), graft insertion into stromal pocket Graft size: 9–11 mm Postoperative medication: chloramphenicol eye drops 0.5% 6x/day (1 month), dexamethasone eye drops 0.1% 4x/day (1 month), followed by FML 0.1% 4x/day, tapered to 1x/day over a period of 1 year
Outcomes	BSCVA (logMAR), BCLVA (logMAR), Kmax (D), Kmean (D), TPT (μm), CCT (μm), complications, success rate

**Table A14:** Quality assessment.

Item	Authors’ Judgement
Consecutive patients?	Unclear
Reasons for inclusion reported?	Yes
Reasons for exclusion reported?	Unclear
Were point estimates and measures of variability presented for the outcome measures?	Yes
Was calculation of statistical power reported?	Yes

**Table A15:** Risk of bias.

Item	Authors’ Judgement
Data-collector blinded?	No
Outcome-assessor blinded?	No
Free of selective reporting?	Yes
Free of other biases?	No

**Table A16:** van Dijk 2014 [27].

Methods	Type of study: Interventional study Study design: Retrospective case series with prospectively collected data
Participants	Country: the Netherlands Number of individuals with keratoconus: 9 Number of eyes: 10 Subgroups: preoperative, 1 month postoperative, 6 months postoperative, latest follow-up Inclusion criteria: contact lens intolerance owing to progressive end-stage keratoconus (K mean ≥ 58D, Kmax steepest ≥ 70D), informed consent Exclusion criteria: -
Interventions	Donor tissue: artificial anterior chamber, epithelial removal (surgical spears), superficial incision (30G needle), peeling Bowman layer (custom-made stripper, DORC International), ethanol 70% Surgical technique: scleral frown incision (5 mm length, 1–2mm outside limbus), mid-stromal pocket (Melles spatula set), glide (Visitec Surgical Glide), air removal, 70% ethanol, trypan blue, Bowman layer placement on the glide, graft insertion into stromal pocket, BSS in anterior chamber Graft size: 9–11 mm Postoperative medication: chloramphenicol eye drops 0.5% 6x/day (1 month), dexamethasone eye drops 0.1% 4x/day (1 month)
Outcomes	BSCVA (logMAR), BCLVA (logMAR), mean anterior simulated keratometry value (D), K max (D), mean posterior keratometry value (D), maximum corneal power (D), central corneal thickness (μm), thinnest point thickness (μm), endothelial cell density (cells/mm^2^), contact lens tolerance

**Table A17:** Quality assessment.

Item	Authors’ Judgement
Consecutive patients?	Unclear
Reasons for inclusion reported?	Yes
Reasons for exclusion reported?	Unclear
Were point estimates and measures of variability presented for the outcome measures?	Yes
Was calculation of statistical power reported?	Yes
	(1)

**Table A18:** Risk of bias.

Item	Authors’ Judgement
Data-collector blinded?	No
Outcome-assessor blinded?	No
Free of selective reporting?	Yes
Free of other biases?	No

**Table A19:** Van Dijk 2015 [31].

Methods	Type of study: Interventional study Study design: Retrospective case series with prospectively collected data
Participants	Country: the Netherlands Number of individuals with keratoconus: 19 Number of eyes: 22 Subgroups: preoperative, 1 month postoperative, 6 months postoperative, 18 months postoperative Inclusion criteria: progressive keratoconus stage III-IV, K max > 67.5D, BSCVA < 20/60, keratoconus progression (Δ simK ≥ 1D ± Δ Kmax ≥ 2D) and visual acuity loss, illegibility for cross-linking/ring segments, informed consent Exclusion criteria: -
Interventions	Donor tissue: artificial anterior chamber, epithelial removal (surgical spears), superficial incision (30G needle), peeling Bowman layer (custom-made stripper, DORC International), ethanol 70%, organ culture medium Surgical technique: mid-stromal pocket, glide (Visitec Surgical Glide), air removal, 70% ethanol, BSS, trypan blue, Bowman layer placement on the glide, graft insertion into stromal pocket, BSS in anterior chamber Postoperative medication: chloramphenicol eye drops 0.5% 6x/day, dexamethasone eye drops 0.1% 4x/day
Outcomes	BSCVA (logMAR), BCLVA (logMAR), mean anterior simulated keratometry value (D), K max (D), mean posterior keratometry value (D), central corneal thickness (μm), thinnest point thickness (μm), endothelial cell density (cells/mm^2^), refraction (D), intra-operative and postoperative complications

**Table A20:** Quality assessment.

Item	Authors’ Judgement
Consecutive patients?	Unclear
Reasons for inclusion reported?	Yes
Reasons for exclusion reported?	Unclear
Were point estimates and measures of variability presented for the outcome measures?	Yes
Was calculation of statistical power reported?	Yes

**Table A21:** Risk of bias.

Item	Authors’ Judgement
Data-collector blinded?	No
Outcome-assessor blinded?	No
Free of selective reporting?	Yes
Free of other biases?	No

**Table A22:** Van Dijk 2018 [37].

Methods	Type of study: Interventional study Study design: Prospective case series
Participants	Country: the Netherlands Number of individuals with keratoconus: 17 Number of eyes with keratoconus: 20 Number of untreated contralateral eyes: 16 Subgroups: preoperative, 1 month postoperative, 1 year postoperative, 2 years postoperative, 3 years postoperative, 4 years postoperative, 5 years postoperative Inclusion criteria: BL transplantation between 2010 and 2012 for progressive keratoconus stages III to IV (progression (simK 1D Δ ± Kmax 2D Δ), follow-up ≥ 5 years, ineligibility for UV-crosslinking or ICRS Exclusion criteria: -
Interventions	Donor tissue: artificial anterior chamber, epithelial removal (surgical spears), superficial incision (30G needle), lifting of BL edge (McPherson forceps), peeling Bowman layer (McPherson forceps custom-made stripper, DORC International), ethanol 70%, organ culture medium Surgical technique: mid-stromal pocket, glide (Visitec Surgical Glide), air removal, 70% ethanol, BSS, trypan blue 0.06%, Bowman layer placement on the glide, graft insertion into stromal pocket, BSS in anterior chamber Graft size 9–11 mm Postoperative medication: chloramphenicol eye drops 0.5% 6x/day (1 month), dexamethasone eye drops 0.1% 4x/day (1 month), followed by FML 0.1% 4x/day, tapered to 1x/day over a period of 1 year
Outcomes	BSCVA (LogMAR, Snellen), BCLVA (LogMAR, Snellen), pachymetry thinnest point (μm), pachymetry central point (μm), Kmax (D), Kmean (D), densitometry 0–12 mm (GSU), endothelial cell density (cells/mm^2^), complications, success rate at 5 years, progression of untreated contralateral eyes

**Table A23:** Quality assessment.

Item	Authors’ Judgement
Consecutive patients?	Unclear
Reasons for inclusion reported?	Yes
Reasons for exclusion reported?	Unclear
Were point estimates and measures of variability presented for the outcome measures?	Yes
Was calculation of statistical power reported?	Yes

**Table A24:** Risk of bias.

Item	Authors’ Judgement
Data-collector blinded?	No
Outcome-assessor blinded?	No
Free of selective reporting?	Yes
Free of other biases?	No

## Appendix C. Search Strategy

### Appendix C.1. Search Strategy for Medline (OVID)

01. “Keratoconus” [Mesh]

02. keratocon*

03. 1 or 2

04. “Bowman Membrane” [MeSH]

05. Bowman membrane

06. Bowman layer

07. 4 or 5 or 6

08. “Corneal Transplantation” [Mesh]

09. transplant*

10. cornea* transplant*

11. 8 or 9 or 10

12. 7 and 11

13. 3 and 12

### Appendix C.2. Search Strategy for Embase

01. keratoconus.sh.

02. keratocon$.af.

03. 1 or 2

04. Bowman membrane.sh.

05. Bowman membrane.af.

06. Bowman layer.af.

07. 4 or 5 or 6

08. cornea transplantation.sh.

09. transplant$.af.

10. cornea$ transplant$.af.

11. 8 or 9 or 10

12. 7 and 11

13. Bowman layer transplantation.sh.

14. 12 or 13

15. 3 and 14

## Appendix D. PRISMA 2020 Checklist

Section and Topic	Item #	Checklist Item	Location Where Item Is Reported
TITLE			
Title	1	Identify the report as a systematic review.	1
ABSTRACT			
Abstract	2	See the PRISMA 2020 checklist for the Abstracts checklist.	1
INTRODUCTION			
Rationale	3	Describe the rationale for the review in the context of existing knowledge.	1–4
Objectives	4	Provide an explicit statement of the objective(s) or question(s) that the review addresses.	4
METHODS			
Eligibility criteria	5	Specify the inclusion and exclusion criteria for the review and how studies were grouped for syntheses.	4
Information sources	6	Specify all databases, registers, websites, organizations, reference lists, and other sources searched or consulted to identify studies. Specify the date when each source was last searched or consulted.	4
Search strategy	7	Present the full search strategies for all databases, registers, and websites, including any filters and limits used.	Appendix C
Selection process	8	Specify the methods used to decide whether a study met the inclusion criteria of the review, including how many reviewers screened each record and each report retrieved, whether they worked independently, and if applicable, details of automation tools used in the process.	4
Data collection process	9	Specify the methods used to collect data from reports, including how many reviewers collected data from each report, whether they worked independently, any processes for obtaining or confirming data from study investigators, and if applicable, details of automation tools used in the process.	4
Data items	10a	List and define all outcomes for which data were sought. Specify whether all results that were compatible with each outcome domain in each study were sought (e.g., for all measures, time points, analyses), and if not, the methods used to decide which results to collect.	4
	10b	List and define all other variables for which data were sought (e.g., participant and intervention characteristics, funding sources). Describe any assumptions made about any missing or unclear information.	4
Study risk-of-bias assessment	11	Specify the methods used to assess the risk of bias in the included studies, including details of the tool(s) used, how many reviewers assessed each study and whether they worked independently, and if applicable, details of automation tools used in the process.	5
Effect measures	12	Specify for each outcome the effect measure(s) (e.g., risk ratio, mean difference) used in the synthesis or presentation of the results.	5
Synthesis methods	13a	Describe the processes used to decide which studies were eligible for each synthesis (e.g., tabulating the study intervention characteristics and comparing against the planned groups for each synthesis (item #5)).	5
	13b	Describe any methods required to prepare the data for presentation or synthesis such as the handling of missing summary statistics or data conversions.	5
	13c	Describe any methods used to tabulate or visually display the results of individual studies and syntheses.	5
	13d	Describe any methods used to synthesize results and provide a rationale for the choice(s). If meta-analysis was performed, describe the model(s) and method(s) used to identify the presence and extent of statistical heterogeneity, as well as the software package(s) used.	5
	13e	Describe any methods used to explore possible causes of heterogeneity among the study results (e.g., subgroup analysis, meta-regression).	-
	13f	Describe any sensitivity analyses conducted to assess the robustness of the synthesized results.	-
Reporting bias assessment	14	Describe any methods used to assess the risk of bias due to missing results in a synthesis (arising from reporting biases).	5
Certainty assessment	15	Describe any methods used to assess certainty (or confidence) in the body of evidence for an outcome.	5
RESULTS			
Study selection	16a	Describe the results of the search and selection process, from the number of records identified in the search to the number of studies included in the review, ideally using a flow diagram.	5 and Figure 2
	16b	Cite studies that might appear to meet the inclusion criteria but were excluded, and explain why they were excluded.	6
Study characteristics	17	Cite each included study and present its characteristics.	Appendix B and Table 3
Risk of bias in studies	18	Present assessments of the risk of bias for each included study.	Appendix B
Results of individual studies	19	For each study, for all outcomes present (a) summary statistics for each group (where appropriate), and (b) an effect estimate and its precision (e.g., confidence/credible interval), ideally using structured tables or plots.	Appendix A
Results of syntheses	20a	For each synthesis, briefly summarize the characteristics and risk of bias among contributing studies.	6–7
	20b	Present the results of all statistical syntheses conducted. If meta-analysis was performed, for each, present the summary estimate and its precision (e.g., confidence/credible interval) and measures of statistical heterogeneity. If comparing groups, describe the direction of the effect.	Appendix A and Table 4
	20c	Present the results of all investigations of possible causes of heterogeneity among the study results.	7–8 and Appendix A
	20d	Present the results of all sensitivity analyses conducted to assess the robustness of the synthesized results.	-
Reporting biases	21	Present assessments of the risk of bias due to missing results (arising from reporting biases) for each synthesis assessed.	Appendix B
Certainty of evidence	22	Present assessments of certainty (or confidence) in the body of evidence for each outcome assessed.	Appendix A and Table 4
DISCUSSION			
Discussion	23a	Provide a general interpretation of the results in the context of other evidence.	8
	23b	Discuss any limitations of the evidence included in the review.	9
	23c	Discuss any limitations of the review processes used.	9
	23d	Discuss the implications of the results for practice, policy, and future research.	9
OTHER INFORMATION			
Registration and protocol	24a	Provide registration information for the review, including the register name and registration number, or the state where the review was not registered.	9
	24b	Indicate where the review protocol can be accessed, or the state where a protocol was not prepared.	9
	24c	Describe and explain any amendments to the information provided at registration or in the protocol.	-
Support	25	Describe the sources of financial or non-financial support for the review and the role of the funders or sponsors in the review.	9
Competing interests	26	Declare any competing interests of review authors.	9
Availability of data, code, and other materials	27	Report which of the following are publicly available and where they can be found: template data collection forms; data extracted from included studies; data used for all analyses; analytic code; any other materials used in the review.	Appendixes A and B
		From: Page MJ, McKenzie JE, Bossuyt PM, Boutron I, Hoffmann TC, Mulrow CD, et al. The PRISMA 2020 statement: an updated guideline for reporting systematic reviews. BMJ 2021;372:n71. doi: 10.1136/bmj.n71 [50]. For more information, visit: http://www.prisma-statement.org/, accessed on 29 November 2022.	
